# Computational Analysis of Whole-Genome Differential Allelic Expression Data in Human

**DOI:** 10.1371/journal.pcbi.1000849

**Published:** 2010-07-08

**Authors:** James R. Wagner, Bing Ge, Dmitry Pokholok, Kevin L. Gunderson, Tomi Pastinen, Mathieu Blanchette

**Affiliations:** 1 School of Computer Science, McGill University, Montreal, Quebec, Canada; 2 McGill University and Genome Quebec Innovation Centre, Montreal, Quebec, Canada; 3 Illumina, San Diego, California, United States of America; 4 Department of Human and Medical Genetics, McGill University Health Centre, McGill University, Montreal, Quebec, Canada; University of British Columbia, Canada

## Abstract

Allelic imbalance (AI) is a phenomenon where the two alleles of a given gene are expressed at different levels in a given cell, either because of epigenetic inactivation of one of the two alleles, or because of genetic variation in regulatory regions. Recently, Bing et al. have described the use of genotyping arrays to assay AI at a high resolution (∼750,000 SNPs across the autosomes). In this paper, we investigate computational approaches to analyze this data and identify genomic regions with AI in an unbiased and robust statistical manner. We propose two families of approaches: (i) a statistical approach based on z-score computations, and (ii) a family of machine learning approaches based on Hidden Markov Models. Each method is evaluated using previously published experimental data sets as well as with permutation testing. When applied to whole genome data from 53 HapMap samples, our approaches reveal that allelic imbalance is widespread (most expressed genes show evidence of AI in at least one of our 53 samples) and that most AI regions in a given individual are also found in at least a few other individuals. While many AI regions identified in the genome correspond to known protein-coding transcripts, others overlap with recently discovered long non-coding RNAs. We also observe that genomic regions with AI not only include complete transcripts with consistent differential expression levels, but also more complex patterns of allelic expression such as alternative promoters and alternative 3′ end. The approaches developed not only shed light on the incidence and mechanisms of allelic expression, but will also help towards mapping the genetic causes of allelic expression and identify cases where this variation may be linked to diseases.

## Author Summary

Measures of gene expression, and the search for regulatory regions in the genome responsible for differences in levels of gene expression, is one of the key paths of research used to identify disease causing genes, as well as explain differences between healthy individuals. Typically, experiments have measured and compared gene expression in multiple individuals, and used this information to attempt to map regulatory regions responsible. Differences in environment between individuals can, however, cause differences in gene expression unrelated to the underlying regulatory sequence. New genotyping technologies enable the measurement of expression of both copies of a particular gene, at loci that are heterozygous within a particular individual. This will therefore act as an internal control, as environmental factors will continue to affect the expression of both copies of a gene at presumably equal levels, and differences in expression are more likely to be explicable by differences in regulatory regions specific to the two copies of the gene itself. Differences between regulatory regions are expected to lead to differences in expression of the two copies (or the two alleles) of a particular gene, also known as allelic imbalance. We describe a set of signal processing methods for the reliable detection of allelic expression within the genome.

## Introduction

In a diploid cell, each gene is present in two copies. The vast majority of microarray-based or RNA sequencing-based gene expression studies do not distinguish between the two copies and measure the sum of the expression of the two alleles. This hides the fact that the two alleles are not necessarily expressed at equal levels, a phenomenon called allelic imbalance (AI) [1]. The complete shut down of one allele results in monoallelic expression (ME). The most drastic example of ME is X-chromosome inactivation, where, in females, one of the two copies of the X chromosome is inactivated and packaged into heterochromatin [2]. Less drastic is random monoallelic expression, whereby a randomly selected copy of a gene or chromosomal region is silenced by epigenetic mechanisms (e.g. methylation). In contrast, imprinting results in parent-of-origin specific inactivation of the maternal or paternal allele, depending on the locus. While monoallelic expression completely silences one of the two alleles, less drastic allelic expression differences can result from a heterozygous 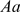 regulatory site. For example, allele 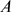 of a transcription factor binding site may allow binding and result in normal expression of the target gene on that chromosome, while allele 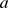 may disrupt the binding site, resulting in lower expression. While the lower expression of allele 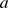 may be compensated by an increased transcription rate at allele 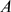 in heterozygous individuals, this may not be the case for individuals who are homozygous 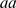, which may result in phenotypic variation. Researchers have tried to identify causative regulatory variants by measuring the total expression (i.e. expression of both copies) of a particular gene across multiple individuals, treating this as a Quantitative Trait Locus (eQTL), and mapping nearby cis-regulatory regions to the gene expression (reviewed in [3]). A key problem with this type of approach is that environmental differences across individuals can affect gene expression, making the mapping problem very challenging.

Instead, a focus on the relative expression of two alleles within the same cell has been suggested to factor out environmental sources of variation, allowing for more sensitive and specific detection of epigenetic and genetic phenomena related to local control of gene expression [4]. Combining AI measurements obtained from a set of individuals with genotyping information about these same individuals, one can map cis-regulatory variants [5]–[8] or detect epigenetic variation in allelic expression [9], [10].

Past studies with the goal of detecting AI have typically relied upon panels of SNPs with relatively low density, located in only a subset of transcribed genes of the genome [10]–[12]. A simple threshold for the ratios of expression of the two alleles at a heterozygous locus is usually established (e.g. 1.5 or 2-fold) and a gene is called as imbalanced based upon whether or not the SNP(s) within it exceed this threshold. Optimal AI profiling in a genome-wide manner would require high-density sampling of expressed heterozygous sites in the genome. We recently generated the first large-scale, high-resolution assay of allelic expression [13]. In this study, Illumina genotyping arrays were used to measure differential allelic expression at 755,284 polymorphic sites in lymphoblastoid cell lines (LCL) derived from 53 CEU samples included in the HapMap project [14]. Because of the noise in single point AI measurements made at each heterozygous locus, sophisticated analytical methods are required to make the most out of this data. In this paper, we develop signal processing approaches for the accurate identification and delineation of transcripts with allelic imbalance, either in a single individual at a time, or in a collection of samples.

To our knowledge, no hypothesis-free computational approaches have been proposed for the analysis of this type of data. Detection of AI in Ge *et al.* [13] relied heavily upon RefSeq, Vega, and UCSC gene annotations, and SNPs were first partitioned into windows corresponding to these annotated regions as well as intergenic regions and windows with significant AI were reported. Sophisticated bioinformatics approaches have been developed for a related, but simpler, problem in the past, that of detecting Copy Number Variants (CNV) or Loss Of Heterozygosity (LOH) in cancer cells using array-based Comparative Genomic Hybridization (CGH) [15]–[18] or genotyping arrays [19]–[25]. These include the PennCNV program [26] and the QuantiSNP program [27], that use a Hidden Markov Model related to one of the approaches considered here. However, CNV or LOH regions have properties that make them easier to detect than regions of allelic imbalance: (i) the signal, coming from genomic DNA is generally quite strong, whereas gene expression can be very low; (ii) the number of copies of an allele is a small integer, whereas the allelic expression ratio is a real number; (iii) the regions affected are typically quite large, whereas AI can affect a single, short gene, or even only part of a gene. The approaches listed above are thus not easily applicable to the detection of AI in gene expression. An alternate family of statistical approaches called changepoint methods has been proposed for segmenting array CGH data into regions exhibiting consistent signals [28], [29]. These non-parametric, model-free approaches have the benefit of segmenting real-numbered data without enforcing discretization. However, they are difficult to generalize to a situation like ours, where signals come from a mixture of discrete (sites with no expression, sites with expression but no imbalance) and continuous (sites with real-valued imbalance) state space.

In this paper, we introduce a family of signal processing approaches for the analysis of AI data obtained from genotyping arrays. We consider both statistical approaches (Z-score computation) and machine learning approaches (Hidden Markov Models) to identify transcripts that show AI and to quantify the latter. We introduce a new type of left-to-right HMM for the joint prediction of allelic imbalance in the 53 samples considered. Our algorithms are evaluated using permutation testing and succeed at identifying regions with known AI. Our approaches reveal that more than 25% of transcripts (coding or non-coding) are subject to differential expression between the two alleles and that patterns of AI are varied and complex. The tools and data sets described here will help biologists and geneticists to identify regions of allelic imbalance, understand the mechanisms at play, identify the genetic or epigenetic causative agents, and associate expression polymorphisms with disease susceptibility.

## Methods

### Allelic Imbalance Data

Allelic imbalance was assayed using Illumina Infinium Human1M/Human1M-Duo SNP bead microarrays. These arrays, originally designed for genotyping, have probes for approximately 1.1 Million polymorphic sites from HapMap, of which 755284 where used for this study. Each probe estimates the abundance of each of the two possible alleles in the sample. Normally, genomic DNA is hybridized onto the chip and the genotypes are easily inferred from the probe intensities. We have previously described how one can take advantage of this technology to measure allelic expression in a high-resolution, genome-wide manner [13]. Briefly, total RNA is extracted and cDNAs are synthesized based on a protocol on heteronuclear RNA, allowing us to measure unspliced primary transcripts [8]. The cDNA sample is hybridized onto the array and each probe estimates the abundance of each of the two alleles in the sample. In parallel, genomic DNA from the same cell line is hybridized, which provides the basis for normalization of the cDNA hybridization while providing us with the genotype of each sample. Details for the full process of experimentally obtaining the raw imbalance information, as well as the sample information, can be obtained from [13].

Data obtained from technical replicates show that although the total expression level (sum of RNA abundance in both alleles) measured at a given SNP is highly reproducible (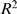 = 0.864), single point allelic expression ratios are much more noisy (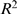 = 0.632), especially for low expression levels (see 9). This suggests that careful data analysis is required to extract as much information as possible.

Let 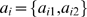 be the set of two alleles present at polymorphic site 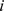 in the population, for 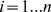 (the rare cases where three or more alleles exist at the same site are ignored in this study). For notational simplicity, we assume that the genome consists of a single pair of chromosomes. In reality, the analysis that follows is repeated separately for each autosome. Genotype phasing consists of the decomposition of the genotype of an individual into its two homologous chromosomes. For individual 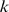, let 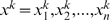 and 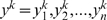, be these two chromosomes, where 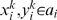. Phasing remains a computationally and statistically challenging problem [30]. In the case of HapMap individuals, phased genotypes are available, although they are not error free. Removal of SNPs not phased in CEU HapMap release R22 resulted in 755284 SNPs which were utilized in our study.

Let 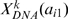 and 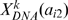 be the intensity read outs obtained from the probes interrogating site 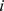 when hybridizing the genomic DNA of individual 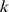. If individual 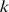 is heterozygous at site 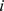 (i.e. 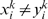), then we expect both 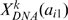 and 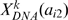 to be large. When it is homozygous, say for 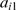, (i.e. 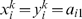), we expect 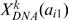 to be large and 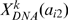 to be small. The genotype of an individual can thus be deduced from the ratio of the two measurements.

Consider now 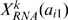 and 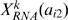, the intensity read outs obtained from the probes interrogating site 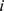 when hybridizing cDNA obtained from whole cell RNA extraction. When heterozygous site 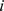 sits in a transcribed region with no allelic imbalance, both 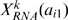 and 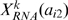 will be relatively large. Any difference between the two may indicate allelic imbalance. Regions that are not transcribed will obtain low values for both alleles. We consider the following pair of observations at each site 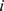:
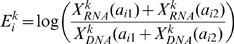
measures the total transcript abundance, and
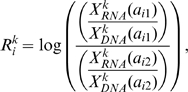
which measures the fold imbalance between the expression of the two alleles. Normalization with the DNA sample, which, for heterozygous sites, is known to be balanced, normalizes for probe sensitivity and biases.

**Figure 1:**
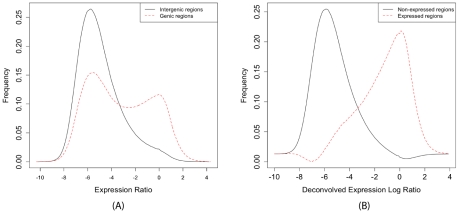
Distribution of 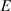 values. (a) Distribution over genic/intergenic regions (b) deconvolutions to expressed/non-expressed regions.

Values for 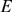 and 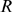 were collected at 755284 sites. Those sites are not uniformly distributed in the genome, with genic regions (exonic and intronic) having roughly 1.3 times the SNP density as intergenic regions (one SNP per 3.5 kb in genic regions, one SNP per 4.5 kb in intergenic regions). Figure 1(a) shows the distribution of 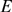 over all genic and intergenic positions. The distribution of expression levels in gene regions is clearly bimodal: a good fraction of genes are not transcribed in LCL, and most but not all intergenic sites are not transcribed. Assuming that 50% of genes and 10% of intergenic sites are expressed, we can deconvolve these distributions to obtain the distribution of 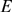 for expressed and non-expressed regions (Figure 1(b)). For two individuals, experiments were done in triplicates. As seen in Figure S1 (a) and (b), the technical noise in the measurement of both 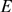 and 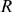 is quite significant. As expected, 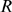 values are particularly noisy at low expression levels.

### Identification of Transcripts with Allelic Imbalance

The main problem addressed in this study is the statistically robust identification of genomic regions with significant and consistent allelic imbalance. We start by noting that the data is too noisy to accurately call imbalance based on each SNP individually (e.g. by simply using on 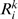), especially for regions whose expression level is relatively low. We thus consider approaches that take advantage of the fact that most regions with AI are relatively long and are expected to contain more than one SNP. Four main approaches were designed, implemented and compared. Each method aims to robustly assign a score 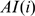 to each SNP 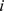, so that SNPs that belong to transcripts with significant allelic imbalance obtain large (positive or negative) scores. In all our AI detection algorithms, AI is detected without reference to any kind of gene annotation, contrasting with the annotation-driven approach used by Ge *et al.* [13], which allows us to identify regions of AI whose boundaries does not necessarily correspond to annotated genes. The first three approaches consider data from each sample individually while the last considers data from all samples jointly in order to improve the detection of AI in individual samples. The four approaches considered are first summarized below and then described in details. The code implementing each algorithm is available at http://www.mcb.mcgill.ca/~blanchem/AI/code.zip.

- **Simple smoothing** refers to the approach where the allelic imbalance log-ratio of a SNP is taken as the average of its own log-ratio and that of the 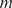 surrounding SNPs on either side.
- **The Z-Score approach** involves binning SNPs based on their expression level, assigning each SNP a Z-Score based on its own allelic imbalance ratio, and then determining the Z-Scores of windows of consecutive SNPs and assigning this score to each SNP within the window.
- **The ergodic HMM approach** models the AI data in a given individual as being generated by a Hidden Markov Model whose states correspond to different levels of total expression and allelic ratios.
- **The left-to-right HMM approach** is an extension of the ergodic model that allows using the AI data from all individuals in order to assess the frequency of AI at each site, and then use those as site-specific priors on the transition probabilities to predict AI regions separately for each individual, but in the context of the data from other individuals.

### Simple Smoothing Approach

Consider heterozygous site 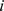 and define window W(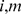) to be the set consisting of 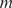 heterozygous sites to the left of 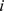, 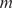 heterozygous sites to the right of 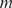, and 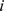 itself. The simple smoothing approach estimates 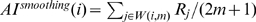. Any site 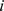 with 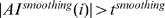 would then be reported as having imbalance, for some appropriate threshold 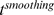. Based on False Discovery Rate assessment (described below), a value of 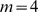 was determined to be the optimal window size and was used for all results reported.

### Z-Score Approach

At sites with no allelic imbalance, the value of 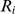 is modeled adequately using a normal distribution centered at 0. However, the variance is inversely correlated with the total expression 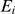, as AI is difficult to estimate when the total expression is low (see Figure S1b). The range of possible values of 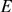 are subdivided into 100 bins of equal size and the mean 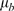 and variance 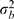 of 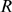 values were determined for SNPs belonging to every expression level bin 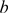. A site-specific Z-Score 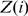 is assigned to heterozygous site 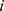 as 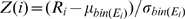. Homozygous sites, being uninformative with respect to allelic ratios, are excluded from the analysis. Consider now a collection of 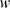 consecutive heterozygous (ignoring possibly intervening homozygous sites) SNPs 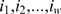. We define the regional Z-score as 
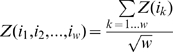
. Assuming the normality of noise in 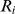 measurements, 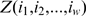 follows a Normal(0,1) distribution under the null hypothesis of absence of allelic imbalance.

Regional Z-Scores are first computed for every possible window of 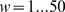 heterozygous sites. The region with the highest regional Z-score (in absolute value), 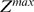 is selected first and we set 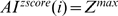 for all sites heterozygous 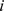 within the region. This region is then masked out and the next highest scoring non-overlapping window is selected. The process is repeated until all heterozygous sites have a Z-Score assigned. We note that because the 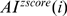 is obtained based on the *best* window that contains site 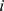, there is an complex issue of multiple hypothesis testing that makes that this measure will not follow a Normal(0,1) distribution under the null hypothesis (i.e. absence of AI). In consequence, one cannot easily translate 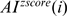 into a p-value.

We also considered a variant of the Z-Score approach where each SNP is assigned the Z-Score of the *fixed-size* window centered around it. This approach, which can be seen as an improved version of our simple smoothing approach, indeed improves on the latter (based on permutation testing and comparison to transcripts with known AI - see below), but is far from being as accurate as the proposed Z-Score approach, because it leads to bleeding edges at transcript boundaries. We also investigated a version of the Z-Score approach where SNPs are not binned by expression level prior to Z-Score computation; this resulted in a small but significant decrease in accuracy, showing that the appropriate modeling of the dependency between the noise in allelic ratio and the total expression level is an important feature of our approach.

### Single-Sample Ergodic Hidden Markov Model Approach

The linear nature of the data in question lends itself well to a Hidden Markov Model (HMM) in which each data point corresponds to a particular SNP, the hidden states correspond to qualitative descriptions of the allelic imbalance (e.g. positive imbalance, negative imbalance, no imbalance), and emissions correspond to the total expression 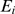 and the allelic log-ratio 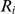 observed at site 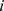.

**Figure 2:**
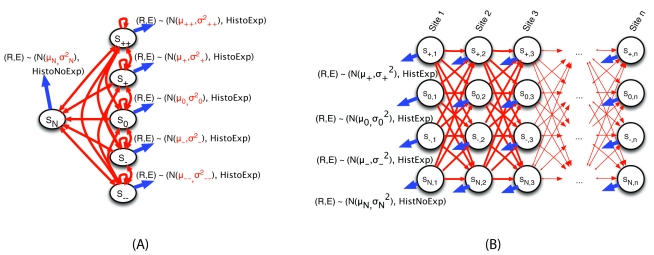
Architecture of the two Hidden Markov model used in this study. (a) Ergodic HMM architecture. HistoExp and HistoNoExp refer to the distributions depicted in Figure 1(b). For readability, states 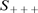 and 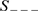 are not shown. (b) Multi-sample left-to-right HMM architecture. States 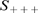, 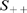, 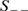, and 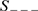 are not shown for clarity. Only transition probabilities are trained. All copies of a given state have the same emission probability distribution, described on their left.

We built an HMM consisting of a total of eight hidden states (see Figure 2a). Seven of these states correspond to SNPs take belong to expressed transcripts in the LCL sample in question, with various levels of imbalance: 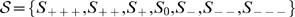, corresponding to strongly positive imbalance (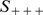), moderately positive imbalance (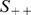), slightly positive imbalance (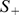), balance (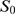), slightly negative imbalance (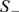), moderately negative imbalance (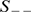) and strongly negative imbalance (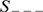). There is also a state (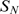) that corresponds to SNPs located in regions that are predicted not to be transcribed, and for which allelic imbalance is meaningless. The emission probability for each state 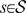 is modeled with a pair of normal distributions for the 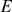 and 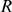 values, with parameters (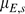, 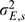), and (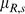, and 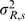) respectively. Whereas both total expression 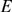 and allelic imbalance measurements 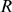 are observed at heterozygous sites, only the expression is measured at homozygous sites. In the latter case, the imbalance data is left unobserved (i.e. all 8 states are equally likely to have generated the 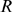 observation). Homozygous SNPs can thus be included in the model training and predictions, and can help delineating regions of based on expression levels.

An HMM with a realistic correspondence to the data can in principle be built with 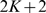 states, where 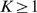 represents the number of levels of positive (and negative) imbalance that the model represents. Larger values of 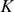 should in principle be favorable as they allow a finer discretization of allelic ratios. Models with 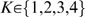 were trained and the false discovery rate measured and compared (see section 0). It was found that 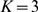 performed better than 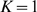 and 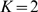, and similarly to 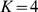 (Figure S2), so this value was used for both the ergodic and left-to-right models.

Certain parameters of the HMM are trained using the Baum-Welch algorithm, while others are fixed. For 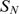, the emission probability distribution for 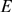 is modeled non-parametrically by the histogram of Figure 1(b) (black curve) whereas all expressing states share the same total expression distribution from Figure 1(b) (red curve). These emission probability distributions are kept constant during the training procedure. The Baum-Welch algorithm [31] is used to find maximum likelihood estimators for 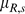 and 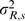, for 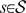, as well as all transition probabilities and the initial state probability. The Baum-Welch algorithm is an expectation-maximization (EM) [32] approach that alternates between the Expectation step (or E-step), in which the posterior probability over states is computed for each site using the Forward-Backward algorithm, and the Maximization step (or M-Step) where the parameters of the emission and transition probability distributions are adjusted to best reflect the observed data given these posterior probabilities. Formulas for updating the emission probability parameters and transition probabilities are adapted straightforwardly from Mitchell [33]. We considered training one HMM per individual (which would allow the flexibility to model inter-experiment variation in noise, for example), or to train a single HMM based on the data from all individuals (which would have the benefit of being based on more data). The latter option produced slightly better results and this is the strategy we used for the rest of the study. We also considered filtering out sites with low total expression, as their allelic expression ratio may be less reliable. However, slightly better results were obtained without any filtering (allowing non-expressed SNPs to naturally be classified as belonging to state 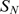). Training on the whole data set took less than Baum-Welch 20 iterations and 3 hours to converge on a standard desktop computer (convergence is defined as two consecutive iterations where no parameter or transition probability changed by more than 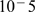 or 1% of their value). Restarts from different initial values converged to nearly the same values.

The Viterbi algorithm [34] can then be used to identify, in each individual, predicted regions of different levels of positive or negative imbalance. The Forward-Backward algorithm [35] yields an estimate of the posterior probability of each state at each site. In the latter case, a useful summary score for each site is the posterior expected allelic expression log-ratio, which we use as AI predictor: 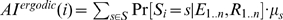.

Until now we have assumed homogenous transition probabilities, regardless of the distance in base pairs between consecutive SNPs along the chromosome. However, a more accurate model would factor in the distance between neighboring SNPs, to increase the probability of self-loops (i.e. staying in the same state) when the two sites are nearby but increase the probability of state change for two distant sites. Such an approach has been used previously in HMMs designed to detect CNVs [27]. We obtained a unit transition probability matrix 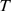 as the 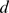-th root of the transition matrix obtained via Baum-Welch training of the homogeneous model, where 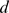 is the average distance (in base pairs) between two consecutive SNPs in our data. Then, the transition probability matrix used for a pair of sites separated by 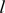 base pairs will be 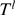, which is efficiently computed using the eigenvalue decomposition of 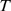.

To ensure that our training procedure was not subject to overfitting, we used 2-fold cross validation (dividing the 53 samples into one 26-sample data set and one 27-samples data set) and trained our 8-state ergodic HMM separately on each half the samples. The parameters and transition probabilities obtained were nearly identical, and so were the FDR estimates obtained by running each HMM on the complementary data set, indicating that overfitting is not an issue.

### Multi-Sample Left-to-Right HMM Approach

The previous HMM is called ergodic because it models an ergodic, homogeneous Markov chain over the state space (i.e. the set of transition probabilities is independent of the position along the genome). One limitation of this HMM is that it does not take full advantage of the fact that data exists for multiple individuals and that, while not all individuals are expected to have AI in exactly the same regions, one does expect AI hotspots where a significant fraction of the individuals would have imbalance. That would be the case, for example, for genes where one allele is commonly or always silenced via epigenetic mechanisms, or when AI is due to a common regulatory variant. The approach proposed in this section aims at predicting AI regions separately in each individual, while taking into consideration the data observed in *all* individuals. In doing so, we still want to be able to identify AI regions that are unique to a given individual, but are hoping to improve the detection of regions with common AI. For example, AI regions containing only a few SNPs, or those where the imbalance is only moderate, may be missed when present in a single individual, but may be detectable if present in a large fraction of the population. In addition, we may be able to detect boundaries of AI regions with more accuracy when they are shared among individuals.

The approach utilized to address this is termed the *left-to-right HMM* [35] (see Figure 2 (b)), similar to profile HMMs [36]. Each site has its own copy of the set of states and transitions can only occur between states associated with neighboring sites, from left to right. Each copy of a given state shares the same emission probability distributions that are modeled the same way as with the ergodic HMM. However, transition probabilities will vary across positions, making the model non-homogeneous (in contrast to our ergodic HMM approach). This configuration allows for greater fine tuning at the level of each individual SNP or region, though at the cost of a substantially larger set of transition probabilities to be learned.

The training of our left-to-right HMM is a two stage process. In the first stage, emission probabilities, transition probabilities, and start probabilities are estimated for the ergodic version of the HMM using the Baum-Welch algorithm described above, using all available individuals. The parameters of the emission probabilities of the states in the left-to-right HMM will be set to those obtained on the ergodic training and will not be re-estimated. The obtained ergodic non-homogeneous distance-corrected transition probabilities will be used as prior for those of the left-to-right HMM.

In the second stage, we now switch to learning the transition probabilities of the left-to-right HMM. We assume that the data set from each individual is the result of an independent run of the HMM: 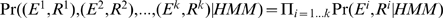, and we seek to identify the set of transition probabilities of the left-to-right HMM that maximizes this joint likelihood. Consider a site 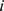 that is not imbalanced in any individual but where site 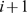 is positively imbalanced in a large fraction of the individuals. The maximum likelihood estimator for the transition from state 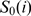 to state 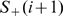 will be higher than at other positions where few individual enter an imbalanced region. Now consider an individual where there is only weak evidence of AI starting at position 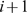. When using an ergodic HMM for our predictions, the weak AI region will probably not be detected. However, in the left-to-right HMM, with the increased transition probability, the AI path becomes more likely, so provided that there is sufficient imbalance, the most likely path may now to go through one of the imbalanced state.

Estimating transition probabilities between two sites separated by 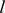 base pairs is done using a simple modification to the standard Baum-Welch algorithm, where the update rule for transitions is: 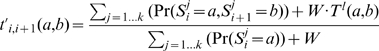 where 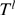 is the 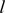-th power of the unit transition probability obtained previously and 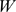 indicates the pseudocount weight described in the following paragraph. The regularization obtained by using the ergodic transition probability as prior reduces the risks of overfitting while improving the convergence of the training procedure. In practice, based upon permutation tests and resulting FDR scores, a parameter of 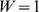 was determined to be optimal (data not shown).

Once the left-to-right HMM is trained using the data from all 53 individuals (which took 161 Baum-Welch iterations - less than 4 hours on a standard desktop computer), the standard Viterbi or Forward-Backbward algorithms are used to identify AI regions separately for each individual. As with the case of the ergodic HMM, we use the posterior expected allelic expression log-ratio 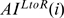 to summarize AI evidence at SNP 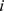.

Overfitting is a possible issue with our left-to-right HMM, as the number of parameters estimated is much larger than for the ergodic HMM. We performed 5-fold cross-validation, training on 4/5 of the data and predicting on 1/5. Thanks to our regularization procedure, the predictions obtained were very similar to those obtained by training and testing on the full data set, with only a marginal decrease in FDR.

### Cross-Hybridization

Upon study of some of the regions where AI was predicted in most or all individuals but where not known imprinted regions existed, we found that nearly half were a likely artifact of cross-hybridization. All these suspicious regions were the results of a segmental duplication, where a fragment of a gene was duplicated. Because the fragments still matches the genic region, sites within them will appear to be expressed (as they match the transcript of the paralogous region), and polymorphisms will cause mismatches between the probe and the true transcript, which will result in apparent AI. We thus used the human Blastz self-alignment from the UCSC Genome Browser [37], [38] to filter out regions corresponding to recent duplications. A possible alternate approach would consist of using the results of the genomic DNA hybridization to identify probes that match more that one location in the genome, with the possible added benefit of detecting DNA possible copy-number variation.

### False-Discovery Rate Estimation

Due to the relatively small number of “gold standard” regions known to exhibit AI, the best available option for comparison of the various models is through permutation tests. The goal was to preserve some of the structure of the genome such that only SNPs with approximately equal expression levels and heterozygosity would be swapped, i.e., the only factor that is swapped freely is that of the allelic imbalance ratio. Permuted data sets were generated as follows. Sites were partitioned into five levels based on the number of individuals in which they are heterozygous. Five bins were also assigned based on the average level of expression seen across all individuals. Each SNP was then finally assigned to one of 25 bins, with one bin for each of the possible combinations of heterozygosity frequency and expression levels. Sites were randomly permuted within each bin, preserving the correspondence between sites in different individuals (in the case of the left-to-right HMM, the first stage of training of global HMM parameters was first done on non-permuted data, and then the second stage of model training was done on permuted data). Preserving expression levels and heterozygosity is important to create permuted data sets that are as realistic as possible, in particular with respect to the fact that expressed sites are found in contiguous genomic regions rather than dispersed randomly in the genome.

Each of the prediction methods described produces one AI score per site and per individual. For each method 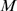, the number of regions of consecutive SNPs exceeding a given score threshold 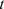, 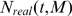 and 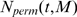 was determined in the real and permuted data, resulting in a False-Discovery Rate of 
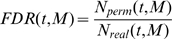
.

## Results

Each of our four approaches was applied to the data set and the AI predictions for each individual are available at http://www.mcb.mcgill.ca/~blanchem/AI/AIPredictions.zip.

### Illustrative Case Studies

**Figure 3:**
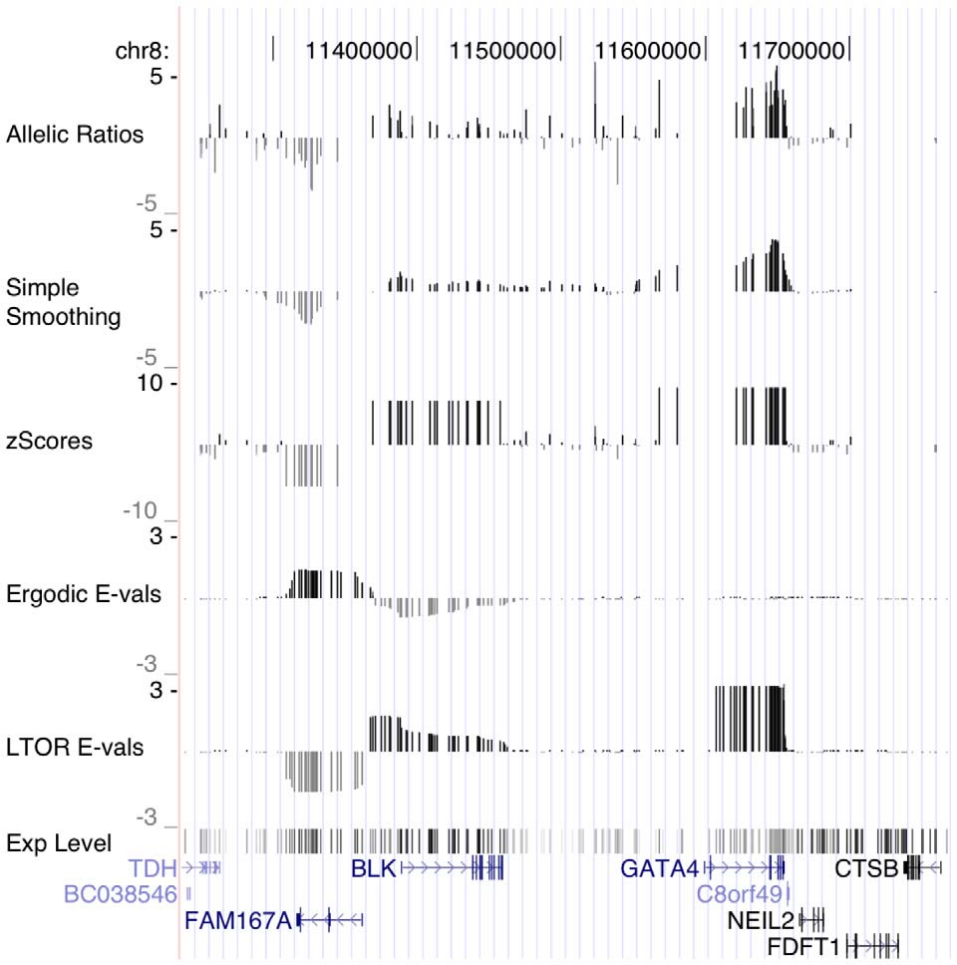
Raw data and predictions. Example of genomic region with allelic imbalance. From top to bottom: Raw allelic log-ratio; Simple smoothing predictions; Z-score predictions; Ergodic 8-state predictions (expected allele log-ratio); Left-to-right 8-state HMM predictions (expected allele log-ratio); Raw total expression; UCSC known genes track. Data shown is for HapMap individual NA11840. Note: Allelic ratios at homozygous sites are not shown.

We use two examples to highlight the features of the data and the methods developed. Figure 3 gives a sample of the raw data and predictions made by each method in the BLK locus. BLK is a gene that has previously been described as allelically imbalanced in LCL [13]. Interestingly, in this individual, two other neighboring genes have strong allelic imbalance, with FAM167A showing expression on the opposite allele compared to BLK and GATA4 also obtaining strong an consistent signals. Although in this example the boundaries of allelic expression domains align nicely with known gene boundaries, this is not the case in general. As is obvious from the figure, the raw expression and allelic ratio data are quite noisy. The simple smoothing approach succeeds at identifying the main regions of allelic imbalance but does so much less reliably and precisely than the other three approaches. Notice that this individual has no heterozygous sites in the 5′ end of FAM167A. This results in different behaviors for each method. The ergodic approach assigns gradually decreasing expected allelic log-ratios in that region, while the Z-Score approach only predicts imbalance in the 3′ end of the gene. However, the left-to-right HMM has the benefit of considering data from other individuals, which have some heterozygous sites in the 5′ region of the gene, which allows it to predict strong and consistent negative allelic log-ratios over the whole gene, and a sharp transition entering the BLK transcript. A similar phenomenon is observed for GATA4.

**Figure 4:**
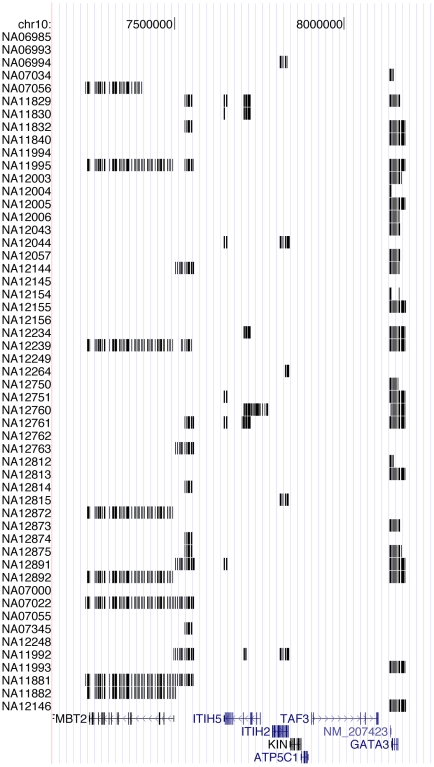
Allelic imbalance in 53 HapMap individual in the GATA3 locus. Each row reports the sites where AI has been predicted by the 8-state left-to-right HMM with the Viterbi algorithm. Each AI SNP is marked with a vertical black line; the impression of gray levels is an artifact of SNP density. Genes from RefSeq [44] are illustrated below.

Figure 4 shows the set of predictions made by the Viterbi algorithm using the left-to-right HMM on the extended GATA3 locus, in all 53 samples. The region exhibits a large diversity of patterns of AI. In some cases, the region of AI closely matches an annotated gene (e.g. SFTMBT2 in several individuals). Often, AI regions do not overlap any known gene (e.g. the region located upstream of SFMBT2). Such regions, especially when they abut an annotated gene, may reflect the presence of alternative allele-dependent promoters. They may also represent completely novel unannotated transcripts. Another frequently observed pattern is the presence of AI within annotated transcripts, near the 5′ or 3′ end (e.g. the 3′ end of the ITIH5 gene). Finally, AI regions often encompass one or more complete genes (e.g. GATA3 and NM_207423), possibly because of epigenetic modification of one of the two alleles. We note based on analysis done in [13] that SFTMBT2 and ITIH5 show evidence of heritable allelic expression, whereas GATA3 does not show correlation with common genetic variants and could represent epigenetic modification of expression in LCLs.

### Evaluation and Validation

The accuracy of the AI predictions made by each method was evaluated using both permutation testing (in order to assess the false discovery rate) and comparison to previously characterized AI transcripts.

### Permutation Testing

**Figure 5:**
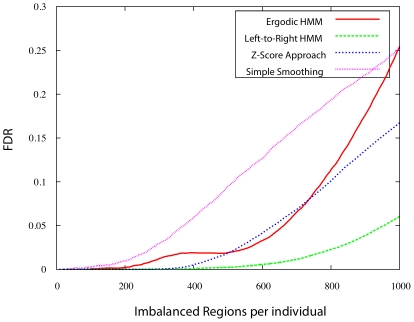
False discovery rates (FDR). obtained by permutation testing at thresholds resulting in different numbers of AI regions being predicted.

We first estimated the false-discovery rate (FDR) of each method using a permutation test where genomic sites are randomly permuted, subject to some constraints (preservation of heterozygosity and expression level; see Methods). This randomized data set preserves the level of imbalance observed at each site, but randomly disperses sites in such a way that few regions are expected to exhibit strong and consistent allelic ratios over several consecutive sites (as real AI transcripts should). For each algorithm, the number of genomic regions with AI score above some threshold 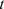 in the real data was compared to the corresponding number on the permuted data - the ratio of these two numbers is an estimate of the FDR of the algorithm (note that the FDR could also be estimated at the individual SNP level, rather than at the region level; the conclusions are the same). Figure 5 shows the FDR curves obtained for each method, as a function of the number of predictions made. All methods are able to detect the most obvious cases of AI (roughly 200 regions per individual, where all methods have near-zero FDR). However, as our threshold decreases and the number of regions predicted increases, the performance of the four approaches become quite different. Setting 5% as an acceptable FDR, the simple smoothing, Z-Score, ergodic HMM, and left-to-right HMMs result in 360, 622, 662, and 954 predicted regions with AI. In other words, at that FDR level, the best approach, left-to-right HMM, is 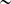
160% more sensitive than the simple smoothing approach and 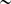
45% more sensitive than the second best approach, which is the ergodic HMM. Similar observations hold for other FDR thresholds. Therefore, the information obtained from the total expression levels, as well as the added site-specific transition probabilities are beneficial in terms of obtaining reliable AI predictions. This is particularly noteworthy for regions whose AI is weaker (those ranking between the 500 to 1000th per individual), for which the FDR remains quite low with the left-to-right HMM but quickly increases with all other methods.

### Comparison to Known AI Transcripts

**Figure 6:**
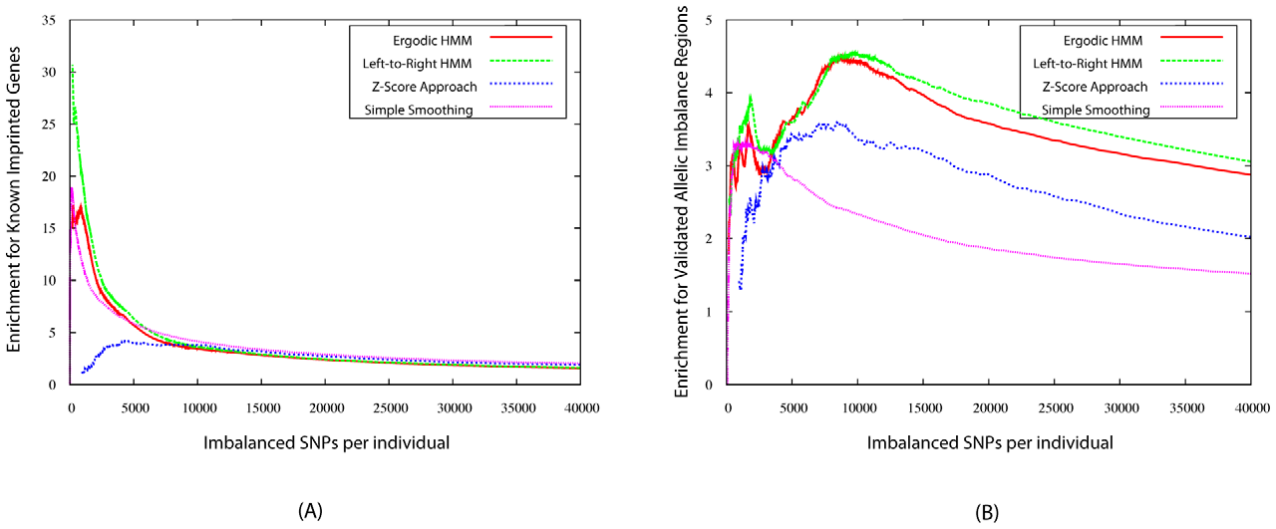
Enrichment for SNPs called as allelically imbalanced in imprinted and AI genes. (a) Overlap with regions experimentally verified to be imprinted. (b) Overlap with experimentally validated imbalanced genes from Verlaan *et al.* [8].

Although no comprehensive set of validated AI transcripts exists to date, a set of 62 imprinted genes (containing 1099 SNPs in our data set) have been collected from the literature and posted on www.geneimprint.com. Most imprinted regions are easily detected by most methods, as they affect relatively large genomic regions and their allelic expression ratios are extremely large. Figure 6 shows how the enrichment of the overlap between imprinted genes and the predictions made by each of the four methods varies as a function of the number of sites being predicted with AI. (The enrichment of the overlap between a set of predicted AI regions and a set of annotated regions is the ratio of the size of the overlap to the expected size of the overlap if AI regions had been selected randomly in the genome.) Imprinted SNPs are enriched 5 to 20-fold among the top predictions made by each algorithm (except the Z-Score approach, which assigns high scores to other types of regions). Focussing on the left-to-right HMM AI predictions at a 5% FDR threshold (which consist of roughly 40,000 SNPs per individual), we find that 67% (resp. 35%) of SNPs in imprinted regions are predicted to have AI in at least one (resp. five) individual. Manual inspection of imprinted genes that have gone undetected by any of our methods reveals genes that are short, contain few heterozygous SNPs, or are expressed at a very low levels in LCL.

Allelic imbalance resulting from cis-regulatory variation typically have allele ratios less extreme than imprinted genes and are thus more difficult to detect. A set of 61 transcripts (containing 1596 SNPs in our data set) with AI resulting from cis-regulatory variation in LCL have been identified and validated by Verlaan *et al.* [8]. Figure 6 (b) shows the fold-enrichment of these SNPs among those predicted as AI SNPs by each of our methods. Here, the predictions made by the two types of HMMs perform significantly better than the Z-Score and smoothing approaches, detecting approximately 50% and 100% more validated SNPs. Overall, our best approach is again the left-to-right HMM, which predicts 87% (resp. 70%) of the 1596 validated SNPS as imbalanced in at least one (resp. five) individual(s). Inspection of AI genes that were undetected showed that they exhibited little evidence of allelic imbalance by our method (see Figure S3). These represent likely false positives in earlier study as well as more localized effects caused by few independent AI measurements and driving the association tests in previous analyses [13].

### Distribution of AI in the Genome and Across Individuals

Our predictions allow a first glimpse into the diversity of allelic expression patterns in the human genome, although a comprehensive analysis of AI regions is beyond the scope of this study. We first observe that AI in LCL samples is widespread, with on average 9.7% (resp. 5.6%) of an individual's genes containing at least one (resp. all) imbalanced SNP (using the left-to-right HMM with a threshold corresponding to an FDR of 5%). Considered in total, 54.4% of genes show at least one imbalanced SNP in at least one individual, and 45.6% of genes have all of their SNPs showing allelic imbalance in at least one individual. Note that only approximately 50% of genes in total are detectably expressed in LCL [39], and hence candidates for being allelically imbalanced. Thus, the majority of expressed genes show AI in one or more individuals.

**Figure 7:**
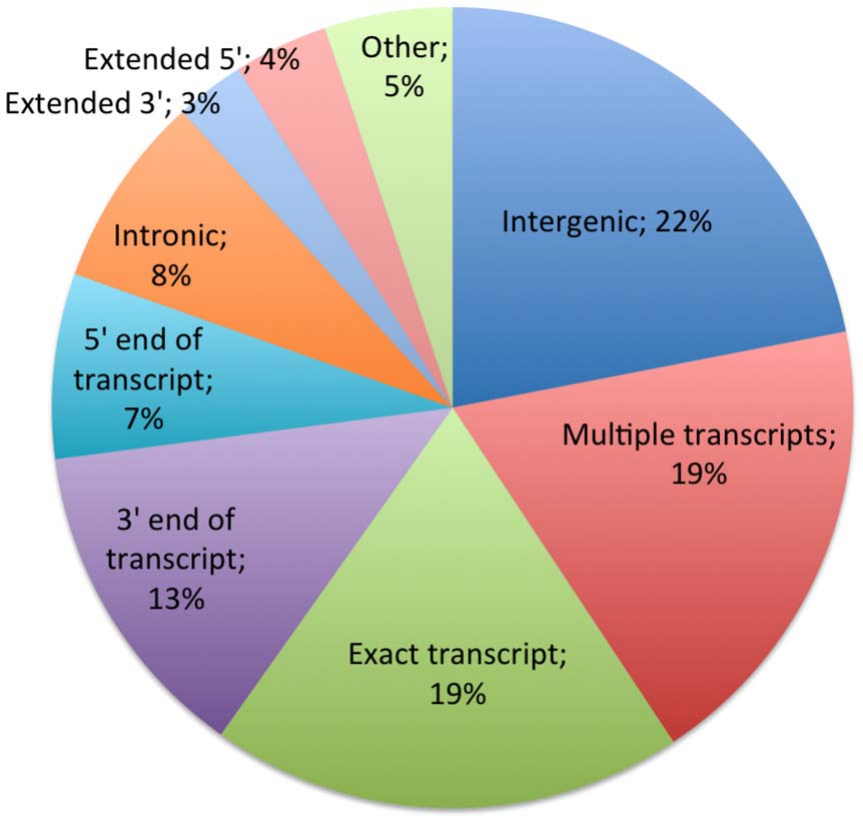
Classification of AI regions based on their overlap with annotated protein-coding genes. The classification of an AI region is done based on a set of simple rules that allow for a sizable margin of error in the boundaries of the AI regions. Intergenic: Little or no overlap with annotated genes. Multiple transcripts: Overlaps several genes. Exact transcript: The left and right boundaries of the AI region match gene boundaries within 20 kb. 5′ (resp. 3′) end of transcript: AI region is at the 5′ end (resp. 3′ end) of the gene only. Intronic: AI region is within the gene but away from the gene boundaries. Extended 5′ (resp 3′): AI region extends upstream (resp. downstream) of the gene.

Figure 7 reports the distribution of AI regions across various types of genomic regions. While a substantial fraction (19%) of AI regions closely match annotated gene boundaries, most exhibit more complex relationships to annotated protein-coding gene transcripts, a larger portion of AI regions (28%) are within annotated genes but cover only a fraction of the transcript. In nearly half of those, allelic expression is found toward the 3′ end of the gene, possibly because of allele-specific transcription termination or mRNA degradation, or the presence of an allele-specific alternate transcription start site within the annotated gene. The presence of AI regions at the 5′ end of the transcript appears somewhat less frequent. 22% have little or no overlap with protein-coding genes, although this fraction is enriched for other types of transcripts such as LINC-RNAs [40].

**Figure 8:**
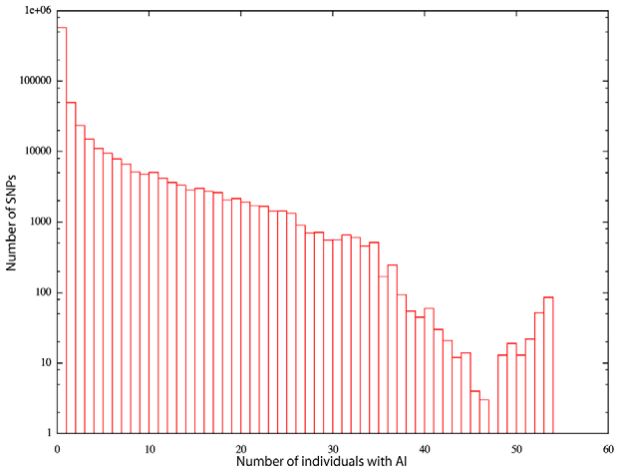
Commonality of allelic imbalance. Number of SNPs in AI regions, as a function of the number of individuals with AI at the same site.

Our data set affords a first glimpse into the commonality of allelic imbalance at a given site across individuals. We calculated the number of individual showing AI (based on the Viterbi predictions; see Figure 8). The very long tail of this distribution indicates that a lot of AI is shared among a portion of the population. In fact, 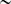
65% of an individual's AI regions are found in at least 10 other individuals. Allelic imbalance, whether caused by genetic or epigenetic causes, is thus highly structured in the human population. On the other hand, rare AI, defined as that seen in at most 10% of our individuals, constitutes approximately 20% of an individual's AI regions, while 4% are unique to that individual. We note however that because AI regions found in a large number of samples are easier to detect than those that are less common in the population, we may underestimate the proportion of AI that is found in a small number of individuals. We note that the left-to-right HMM predictions used for this analysis are potentially biased towards over-predicting sites with common AI and under-predicting those with rare AI. We thus repeated the analysis with the ergodic HMM approach, which does not suffer from this bias. The results were very similar, with only a very slight shift toward less frequent AI.

## Discussion

The recent development of a genome-wide high-density assay of allelic imbalance based on genotyping arrays has resulted in a vast improvement in our understanding of this type of variation and in our ability to map this variation to causative regulatory SNPs [13]. A relatively simple gene-based analysis was sufficient to identify a significant number of genes with allelic imbalance [13]. However, taking full advantage of this technology requires advanced signal processing approaches to accurately detect, delineate and quantify allelic expression. Furthermore, relying too heavily on known gene annotation may hide the fact that most AI does not perfectly align with gene boundaries. Indeed, the approaches proposed here, which do not make use of gene annotations, reveal that allelic imbalance is widespread and exhibits complex patterns in relation to annotated genes. Although our approach was specifically applied to the analysis of data obtained from high-density genotyping arrays, it should be readily applicable to studies based on data obtained next generation RNA sequencing.

Detection of AI based on data from genotyping arrays proves challenging because of the significant noise in the allelic ratio measured at individual SNPs and because of the complex patterns of AI. To our knowledge, our study represents the first in-depth, statistical and computational analysis of a large scale, genome-wide allelic imbalance data set. Because of the noise level in allelic expression ratios at individual SNPs, one must rely on the fact that transcripts with allelic imbalance will generally contain several SNPs that are expected to show imbalance. Our Z-Score approach identifies regions where the allele ratio is significantly different from the expected one-to-one ratio. An aspect of the data that is not exploited by the Z-Score approach is that the total expression and allelic ratio are expected to be consistent across the transcript. Our two HMM approaches model this explicitly, and obtain better results in part because of this. An additional improvement in accuracy of AI detection is obtained by our left-to-right HMM, which considers jointly the data from all individuals to serve as prior for the detection of AI in each one. This approach yields improved detection of AI regions that are shared among many individuals, while being able to detect those present in only one or a few samples. This new type of machine learning problem, where a collection of sequences of observation are expected to have been derived from a common (but unknown) model but where each individual can significantly deviate from that model is a situation that may arise in a number of other situations where our left-to-right HMM approach may be useful, including for comparative genomics based gene predictions [41] (where different species are expected to share some but not all of their exon structure).

Although a detailed biological analysis of allelic imbalance and its phenotypic consequences is beyond the scope of this paper, our predictions reveal that AI is widespread, with roughly 10% of genes showing evidence of AI in a given individual, and with the majority of genes expressed in LCLs showing AI in at least one of our 53 samples. Although roughly 60% of AI regions are clearly related to an annotated transcript, they often reflect the presence of alternative promoters, splicing, or transcription termination.

An increasing proportion of the genetic burden of disease is being associated with differences in gene regulation [42]. At the same time greater complexity of gene regulation and the transcriptome are being uncovered [43]. Therefore, hypothesis-free methods detecting allelic imbalance are a prerequisite to advancing our understanding of population variation in cis-regulatory control by heritable or epigenetic mechanisms.

## Supporting Information

Figure S1 Analysis of the noise using technical replicates. (a) Replicability of expression value *E*. (b) Replicability of allelic ratio *R*.
(0.14 MB TIF)

Figure S2 Performance of ergodic HMM with different levels of discretization. False-discovery rate obtained by ergodic HMMs with 4, 6, 8, and 10 states (corresponding to 1, 2, 3 and 4 levels of positive and negative allelic imbalance).
(0.15 MB TIF)

Figure S3 Analysis of AI data in false-negative regions. Red: Genome-wide distribution of AI measurements (total expression vs allelic ratio). Green: AI measurements in genes identified as imbalanced by Verlaan et al. [8] but not predicted as such by our approach. These genes show no sign of imbalance in our data.
(0.62 MB TIF)
