# Supplementary figures and images for: Computational Analysis of Whole-Genome Differential Allelic Expression Data in Human
Source: PLoS Comput Biol. 2010 Jul 8;6(7):e1000849. doi: 10.1371/journal.pcbi.1000849 (PMC2900287; doi:10.1371/journal.pcbi.1000849)

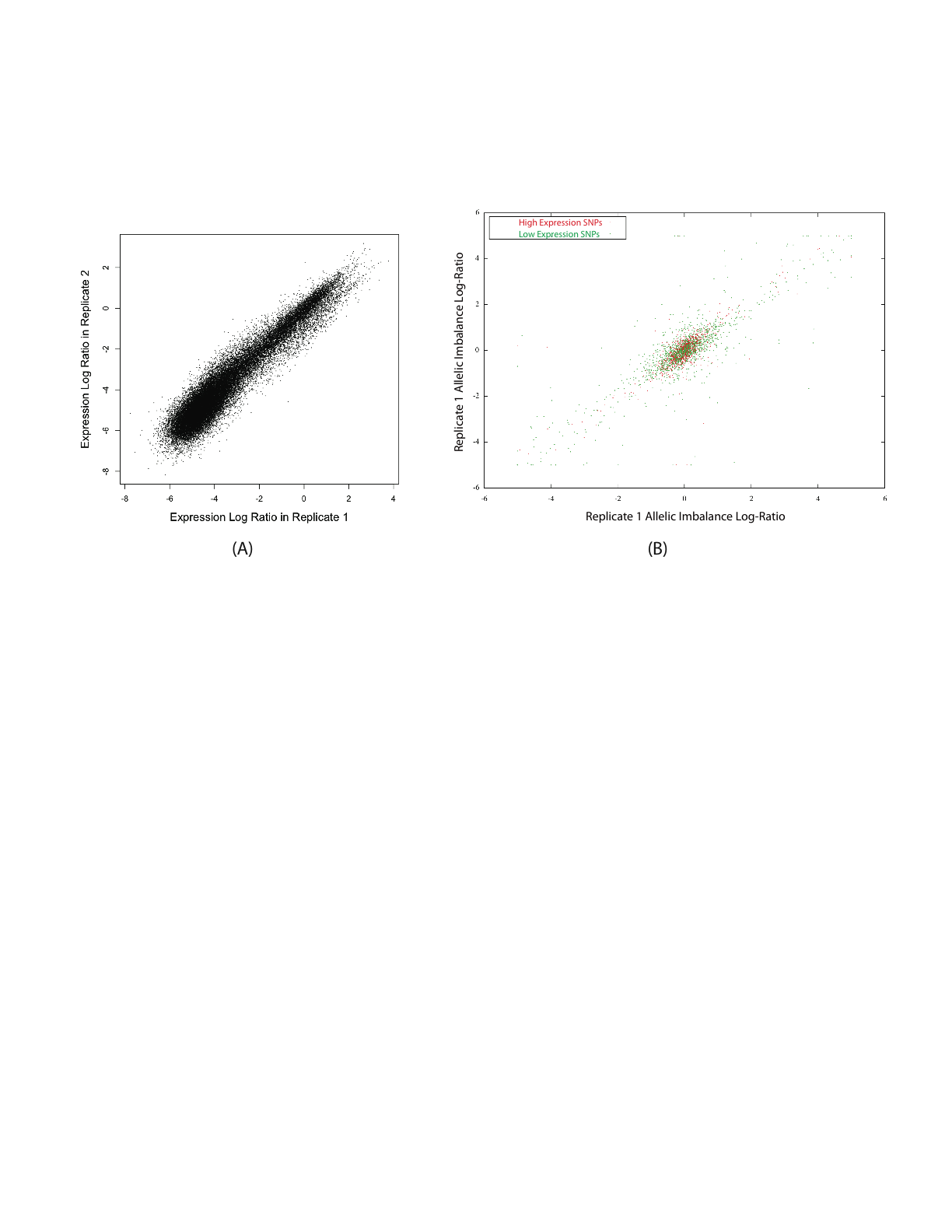

Supplement: Figure S1 — Analysis of the noise using technical replicates. (a) Replicability of expression value E. (b) Replicability of allelic ratio R. (0.14 MB TIF) [file pcbi.1000849.s001.tif]

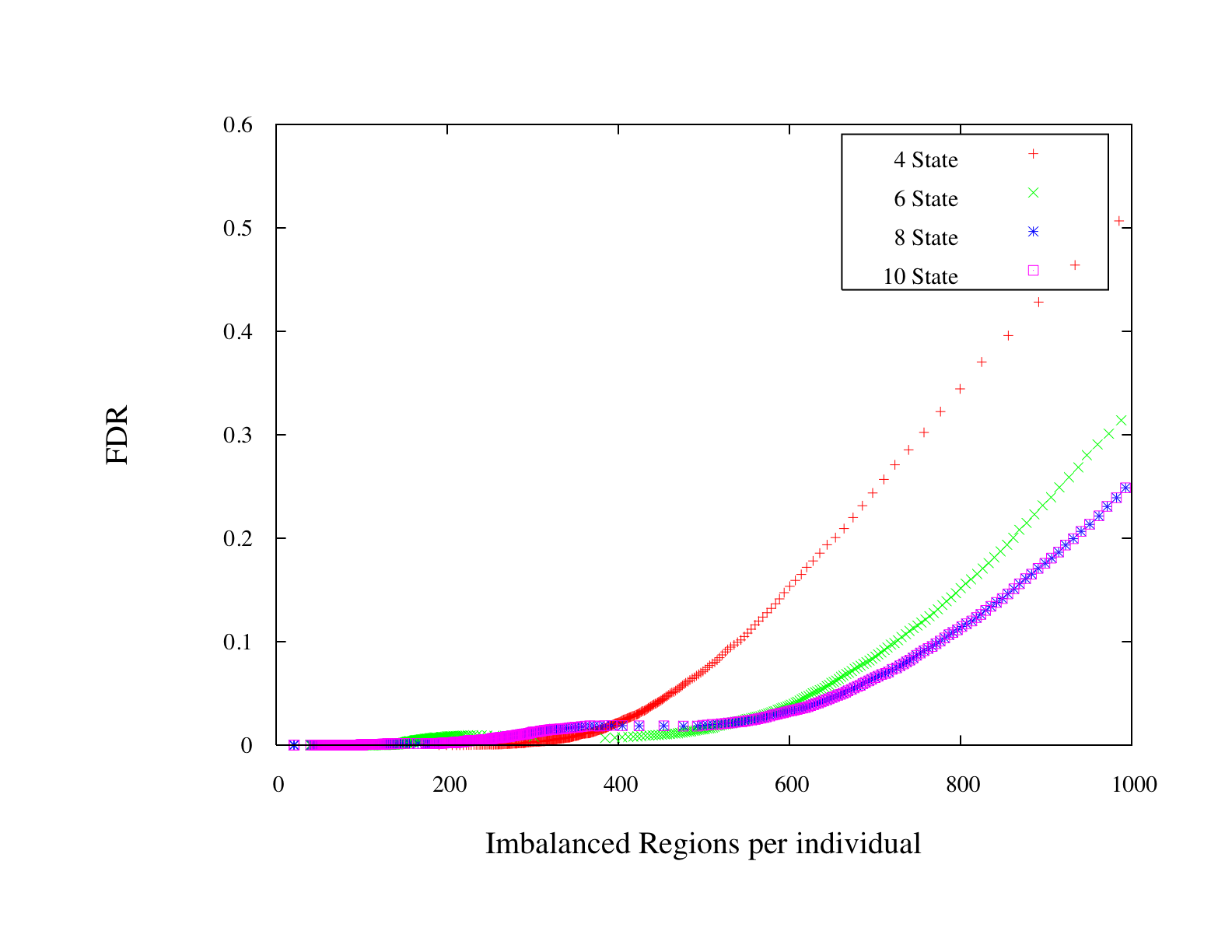

Supplement: Figure S2 — Performance of ergodic HMM with different levels of discretization. False-discovery rate obtained by ergodic HMMs with 4, 6, 8, and 10 states (corresponding to 1, 2, 3 and 4 levels of positive and negative allelic imbalance). (0.15 MB TIF) [file pcbi.1000849.s002.tif]

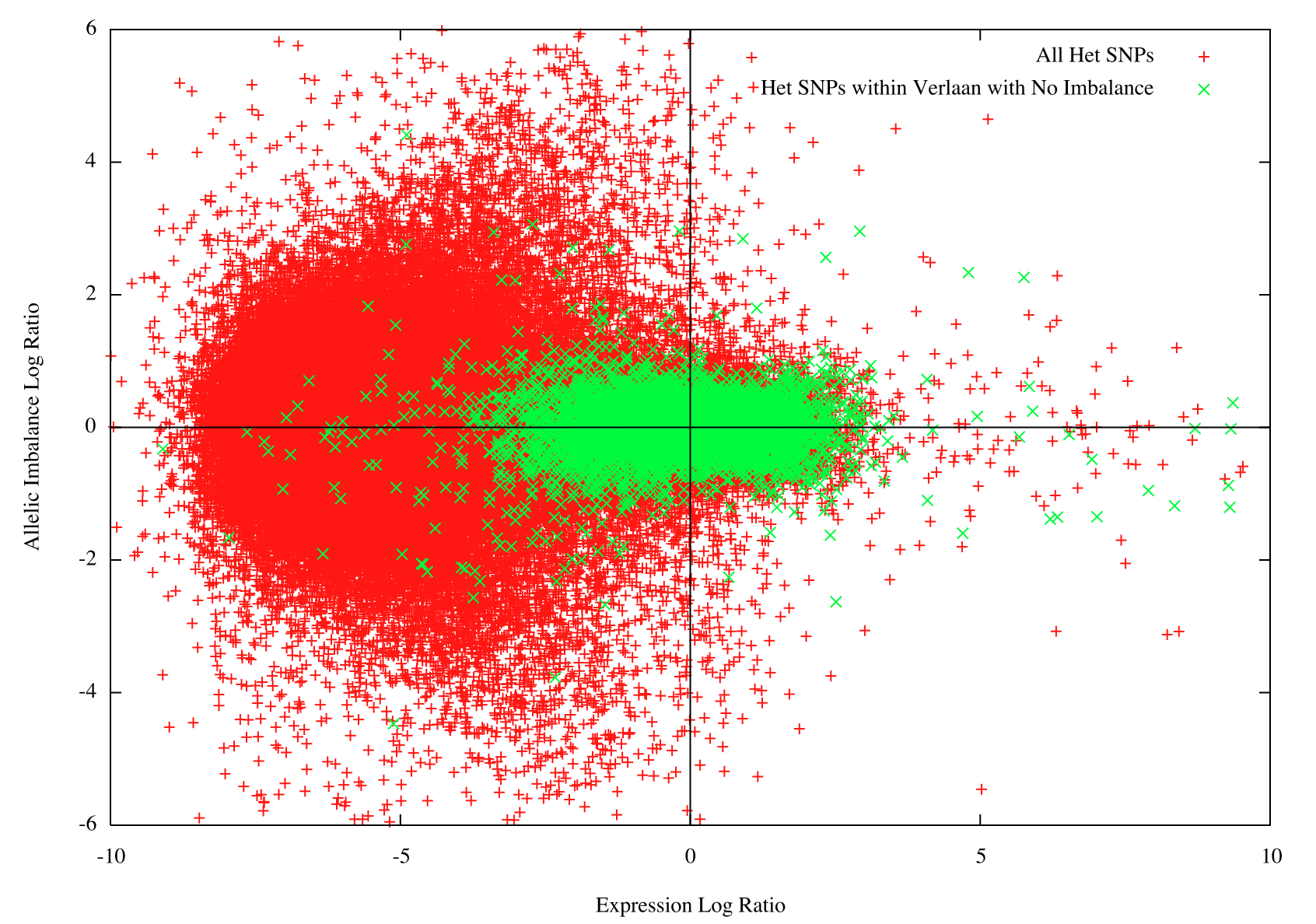

Supplement: Figure S3 — Analysis of AI data in false-negative regions. Red: Genome-wide distribution of AI measurements (total expression vs allelic ratio). Green: AI measurements in genes identified as imbalanced by Verlaan et al. [8] but not predicted as such by our approach. These genes show no sign of imbalance in our data. (0.62 MB TIF) [file pcbi.1000849.s003.tif]
